# Factors affecting resting heart rate in free-living healthy humans

**DOI:** 10.1177/20552076221129075

**Published:** 2022-10-05

**Authors:** Jason Alexander, Magdalena Sovakova, Graham Rena

**Affiliations:** 1 Division of Cellular Medicine, Ninewells Hospital and Medical School, 3042 University of Dundee, Dundee, UK

**Keywords:** Cardiovascular disease, diabetes, digital health, general, exercise, lifestyle, wearables, personalised medicine

## Abstract

Resting heart rate (RHR) is a potential cardiac disease prevention target because it is strongly associated with cardiac morbidity and mortality, yet community-based monitoring of RHR remains in its infancy. Recently, smartwatches have become available enabling measurement with non-intrusive devices of relationships between RHR and other factors outside the laboratory. We carried out cross-sectional observational retrospective analysis of anonymised smartwatch data obtained by participants in their everyday lives between 2016 and 2021 in a single centre community-based study, using convenience sampling. Between participants, overall RHR means strongly or moderately inversely correlated with means of stand hour (SH), calculated VO_2_ max, walking and running distance (WRD), steps and flights climbed (FC). Within participants, in quarterly averages, RHR inversely correlated moderately with frequency of standing (stand hours, SH). RHR also inversely correlated moderately with heart rate variability (HRV), consistent with the known impact of increasing parasympathetic dominance on RHR. These within participant correlations suggest that RHR might be modifiable by changes in SH and HRV within individuals. Indeed, analysing paired daily data, relationships between these three categories were dose dependent. 15 SH versus 5 SH associated with a reduction of 10 beats per minute in mean RHR and increase in mean HRV of 14 ms, respectively. We conclude that within individuals, RHR inversely correlates with frequency of standing and HRV, with paired daily measurements indicating effects are mediated that day. RHR also inversely correlates with fitness and activity measures between participants. Our findings provide initial community-based observational evidence supporting further prospective interventional investigation of frequency of standing or HRV modifiers, alongside more familiar interventions, for cardiac disease prevention.

## Introduction

Numerous lab-based studies have evidenced that higher resting heart rate (RHR) is associated with cardiovascular disease morbidity and mortality^1–5^ but there has been relatively little work done to establish factors influencing RHR in the community. Smartwatches and smart phones are new technologies collecting large longitudinal datasets on VO_2_ max, steps, posture, elevation (measured as flights climbed, FC), walking and running distance (WRD) and heart rate variability (HRV), in the community.^6^ Here, we exploit this data-gathering to investigate for the first time in retrospective observational study, which of these factors correlate with RHR in free-living humans on a day-to-day basis and over quarterly time periods.

Higher RHR is an indicator of increased CVD mortality in apparently healthy individuals and in patients with coronary heart disease (CHD), independently of other major risk factors.^2,7^ RHR is controlled by autonomic nervous system poise (ANS), which can in turn be modulated by many kinds of stress. Increased sympathetic system dominance during stress, which will raise RHR, can itself be measured non-invasively by a decrease in HRV.^8^ HRV is inversely associated with CVD risk.^9^ Previous meta-analysis found that an increase of 1 ms in lab-measured HRV (standard deviation of NN intervals, SDNN) reduces risk of CVD by ∼1%.^9^ An acute decrease in HRV accompanies the shift from parasympathetic to sympathetic dominance during the transition from horizontal to more upright body positions^10,11^; in contrast however, chronic standing has been associated previously with elevated HRV.^12^ Some watches measure frequency of standing throughout the day, termed the Stand Hour (SH), defined as standing with movement for 1 minute in an hour, arguably more akin to a desk break; however, the significance of this measure on HRV and RHR is unknown. In previous work, psychological benefits of desk breaks have been identified^13^ and it is known that physical activity can reduce higher risk of death associated with long sitting time^14^; however, physiological mechanisms remain largely unknown. Further investigation of SH might therefore provide a means to establish a better evidence base for prospective studies investigating desk break interventions.

Cardiorespiratory fitness (VO_2_ max) also inversely correlates with RHR at a population level^1^; however, previous studies have tended to focus on effects of types of activities on RHR rather than how overall activity (measured by WRD, FC, steps) impacts RHR, and how strongly. The purpose of the current study was to determine whether changes in activity, fitness and/or SH correlate with changes in RHR and HRV in the community, within and between participants.

## Methods

### Ethical approval, recruitment, exclusion criteria and data collection

The present study was approved by the University of Dundee School of Medicine Research Ethics Committee (SMED REC Number 20/55). Recruitment of 20 healthy volunteers took place in early 2021. Convenience sampling occurred via social media, word of mouth and email distribution. The sample size was also based on convenience, determined by the time available for the project. Although we were powered to answer the questions we posed for ourselves, more participants would have given more power to stratify the cohort by age, BMI and other factors that might contribute towards the correlations we observed. Recruitment aimed to be balanced in terms of both age and gender, targeting mainly staff and students at the University of Dundee, as well as the Dundee Roadrunners, a local amateur running club. All eligible volunteers who submitted any data to us had it analysed and included in this study. Volunteers had to give written consent and were able to withdraw from the research project at any stage. No consented eligible participants who provided data did withdraw. Exclusion criteria for this study were individuals: (1) outside the age range of 18–60 years, (2) currently taking any prescribed medication or receiving treatment from a medical doctor or (3) currently living outside the Tayside or Fife Health Board areas. In addition, there were technical requirements that would exclude, if the prospective participant was (4) not regularly wearing a watch measuring the categories listed, or (5) with Apple iPhone software not as up to date as iOS 14 or (6) without any of the data categories syncing to the Apple Health application. Watch data was extracted using a third-party app “Health Auto Export to CSV”, which converted the data into a conventional CSV file. Participants were reimbursed for the cost of the app. The CSV file was uploaded by the participant directly to University of Dundee OneDrive secure server in a pre-anonymised manner. Volunteers were asked to export data extending from when they first started regularly wearing their watch until present so that as much data as possible could be analysed. All the analysed data was captured between 2016 and early 2021. The analysed and published data were fully anonymised. Data was not collected for every category from every device. Certain categories, including SH, VO_2_ max and HRV, are either unique to Apple devices, or they are only captured by the phone app if they are collected on an Apple watch. For correlations, we could not include data from an individual if their device had only captured one of the data categories under investigation. We also excluded data values of 0 and for histograms restricted the ranges where there was insufficient data but other than this, all data were included for analysis, both strategies recommended recently by others.^15^ Except in sub-group analysis, each pairwise correlation includes data collected from at least ten participants, as indicated in the tables. A STROBE checklist was followed when planning the manuscript. As far as possible we followed recently published guidelines on research involving wearables.^15^

### Measurements

#### Step count (SC) and stand hour (SH)

Apple watches have been validated previously for SC against manual assessment of steps in a short video recording^16^ and against a reference standard pedometer.^17^ Other studies have validated that other brands of consumer step counter including Garmin perform similarly to an Apple device.^18,19^ Another study validated a Garmin watch against a research-grade pendulum pedometer (Yamax) (Mean Absolute Percentage of Error [MAPE]-4).^20^ SH, a measurement on Apple watches only, represents the number of hours where an individual has stood *and moved* for at least 1 minute. Apple's method of detection for this is not in the public domain but thought to be similar to SC using an accelerometer and gyroscope. To develop an idea of how this related to movement in the community setting, we analysed a 1-month sample of hourly data from one watch. We found that 93% of SH were recorded in an hour where at least one step was recorded and in addition, SH were recorded more often when more steps were recorded. One SH was recorded as follows: No steps in 1 hour, SH recorded 8% of the time *n* = 251; 1–10 steps in 1 hour, SH recorded 22% of the time *n* = 9; 11–50 steps in one hour, SH recorded 62% of the time *n* = 81; 51–100 steps in one hour, SH recorded 95% of the time *n* = 87; 101–250 steps in one hour, SH recorded 97% of the time *n* = 138 and over 251 steps in one hour, SH recorded 100% of the time *n* = 177.

#### Walking and running distance (WRD)

WRD is an estimate of the number of kilometres travelled when walking or running. The watches assess this parameter with the use of global positioning satellite (GPS) communication, which detects their location.^21^ Studies of Apple and Garmin watches have found that they are accurate in recording distances travelled, when compared to trundle wheel reference measurements (MAPE average 2.8)^21^ and in an accurately measured public half marathon.^22^

#### Resting heart rate

Apple watch *RHR* measurements have previously been validated against manual RHR measurements (MAPE 0.07),^23^ an ECG (Concordance Correlation Coefficient (CCC) > 0.9).^24^ Garmin watch heart rate measurement has been validated by correlation with a Polar RS400 chest strap monitor (*r* = 0.997 *p* < 0.0001)^25^ and gold-standard chest strap (gold standard because it corresponds so closely with ECG data).^26^

#### Heart rate variability (HRV)

Measurements from only Apple watches were taken in this study because only these devices record this information in the Health app. Apple watches have been validated against the Polar H7 (CCC 0.989 and 0.977, respectively) in measuring HRV.^27^ The Apple HRV algorithm evaluates the SDNN (standard deviation of the NN interval) HRV measure, which is most sensitive to ultralow and low frequency fluctuations in heart rate,^28^ whereas the ANS system impacts both low frequency and high frequency heart rates.^29^ When measured over 24 hours, SDNN has been inversely correlated with CVD mortality^30^ but instead of measuring over 24 hours, the Apple Health app reports measurements at one or more discrete time points during the day, which we then derived a mean daily value from.

#### Flights climbed (FC)

FC is simply the number of times 10 feet or 3 metres of elevation gain, when walking, running or climbing, is detected by a watch. Apple and Garmin watches measure this using built-in barometric altimeters that detect atmospheric pressure changes in line with their accelerometers and gyroscopes.^31^

#### VO_2_ max

Also known as maximal oxygen uptake, VO_2_ max is the maximal oxygen that a person can utilise during intense exercise. It is viewed as a marker of overall physiological fitness. Repeated longitudinal measurement of VO_2_ max by cardiopulmonary exercise testing (CPET) is impractical to conduct during community-based exercise, which occurs at a variety of intensities.^32^ Calculated VO_2_ max on Apple watches has been validated against CPET in a technical note published on the Apple website. It is unclear if there has been peer review of this data.

For RHR, HRV and VO_2_ max, if more than one value was recorded for a given day, these values were averaged before analysis.

### Statistical analysis

Statistical testing was conducted using the “Statistical Package for the Social Sciences” (SPSS) software, GraphPad Prism, MS Excel and for repeated measures correlation, we used rmcorrShiny.^33,34^ With the exception of VO_2_ max, all daily data were not normal distribution (data not shown), and scatter plots were suggestive of heteroscedasticity for most daily data categories, with curvilinear correlations. We used Pearson's correlation for any data which involved averaging of data over time or between individuals as this resulted in scatter plots with clear linear relationships between variables. The following cut-off values were used to interpret correlations: *r* < 0.20 = very weak; 0.20 to 0.39 = weak; 0.40 to 0.59 = moderate; 0.60 to 0.79 = strong; and 0.80 to 1.0 = very strong.^35^ Error bars on histograms are SEM.

### Data availability statement

Anonymised data supporting the findings of this study may be made available on reasonable request from the corresponding author GR, and where this is consistent with the terms of the ethical approval.

## Results and discussion

### Cohort description

In this study we recruited twenty healthy male and female participants aged 18–60 to study their smartwatch data. The population is described in Table 1. 70% of the cohort was below 30 years old, 60% had a BMI < 25 and 60% were male. All but one of the cohort was not currently smoking. Mean values of measurements we analysed are shown for the whole cohort in Table 2.

**Table 1.** Demographic characteristics of the whole cohort.

Gender	Male	Female	
(*n*)	12	8	
(%)	60%	40%	
**BMI**	**<24.99**	**>25**	
(*n*)	12	8	
(%)	60%	40%	
**Smoking**	**Never**	**No**	**Yes**
(*n*)	11	8	1
(%)	55%	40%	5%
**Age**	**18–29**	**40–49**	**50–60**
(*n*)	14	5	1
(%)	70%	25%	5%

**Table 2.** Mean values for the whole cohort.

	Mean activity measures/day
Steps	8600.8
Walking/running distance (WRD, km)	7.2
Flights climbed (FC)	15.8
	Cardiorespiratory fitness
VO_2_ max (ml/kg/min)	41.0
	Heart measurements
Heart rate variability (HRV, ms)	49.6
Resting heart rate (RHR, bpm)	59.1

### Correlations between RHR and other smartwatch measurements within participants and between participants

We followed guidance on analysis of repeated measures data in a series of short notes by Bland and Altman,^36–38^ to distinguish ‘within participant’ from ‘between participant’ correlations.

#### Between participant correlations

To determine whether a low RHR correlated with any of the other parameters between participants, we carried out correlations of participant means.^37^ We found that RHR inversely correlated moderately or strongly with HRV, SH, VO_2_ max, Steps, WRD and FC (Table 3). VO_2_ max and WRD also both correlated strongly with HRV (Table 3).

**Table 3.** Pearson correlations of participant means.

Pairwise correlations		Correlation coefficient (*r*)	*p-*Value	Participants (*n*)
Data category 1	Data category 2			
Resting heart rate	Stand hours	−0.689	0.028	10
Resting heart rate	Heart rate variability	−0.530	0.051	14
Resting heart rate	VO_2_ max	−0.685	0.010	13
Resting heart rate	Walking and running distance	−0.594	0.020	15
Resting heart rate	Flights climbed	−0.573	0.016	17
Resting heart rate	Step count	−0.489	0.047	17
Stand hours	Heart rate variability	0.287	0.393	11
VO_2_ max	Heart rate variability	0.678	0.008	14
Walking and running distance	Heart rate variability	0.638	0.019	13
Flights climbed	Heart rate variability	0.286	0.302	15
Step count	Heart rate variability	0.491	0.063	15

#### Within participant correlations

When searching for new public health interventions targeting RHR, ideally there would be evidence that changing a variable within a participant, is associated with a change in RHR within that participant. To investigate this, we next performed a repeated measures correlation within participants.^33,36^

**Figure 1.**
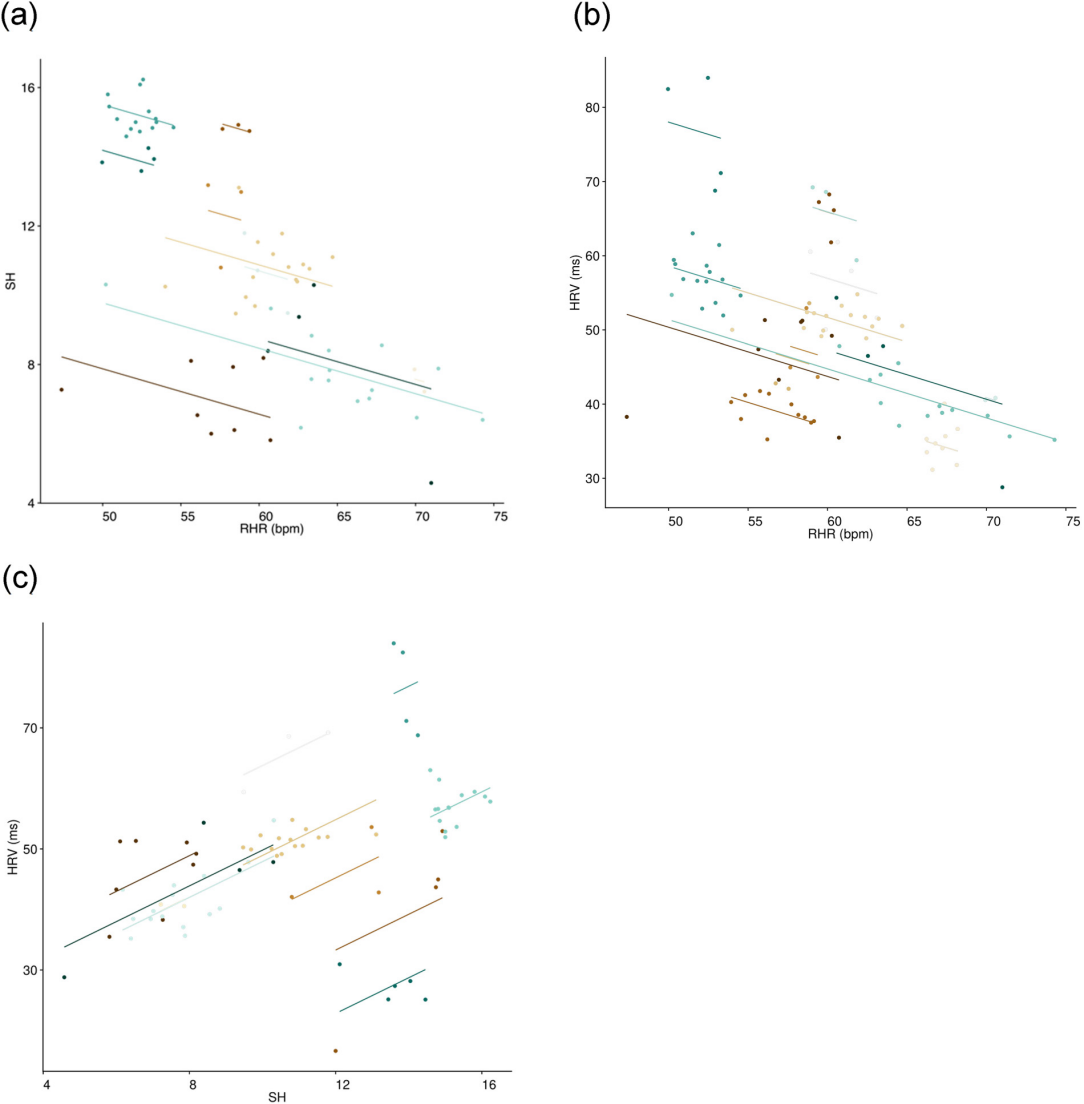
Within individuals repeated measures correlations. Repeated measures correlations were carried out using RMCorrShiny on quarterly averaged data for (a) SH vs. RHR, (b) RHR vs. HRV and (c) HRV vs. SH.

In scatter plots we found that relationships in daily data were curvilinear but that relationships became linear in quarterly paired data. We derived quarterly mean values for RHR, SH, HRV, steps, WRD, FC and VO_2_ max for each individual participant and determined correlations for these data within participants. As in the ‘between participants’ analysis, SH inversely correlated moderately with RHR in the ‘within participants’ analysis (Table 4, Figure 1(a)) and HRV also inversely correlated moderately with RHR (Table 4, Figure 1(b)). One likely mechanism for these correlations is increasing dominance of the parasympathetic system in response to increasing SH. Consistent with this possibility, SH correlated moderately with HRV (Table 4, Figure 1(c)). To test for possible impact of age, sex or BMI, we examined three sub-groups: (a) individuals aged 18–30; (b) males and (c) individuals with BMI < 25. We found that correlation of SH with HRV was maintained in all three sub-groups. Inverse correlation of SH with RHR, as well as HRV with RHR, was also maintained in the first two sub-groups but ablated in individuals with BMI < 25 (Table 4). Although SH/HRV correlation was observed in all groups, these results suggest that SH may be more effective at lowering RHR in overweight people. It is well known that obesity increases dominance of the sympathetic system^39,40^ and it can be speculated that this defect is corrected by increasing SH. VO_2_ max also correlated weakly with HRV but did not correlate with RHR.

**Table 4.** Repeated measures correlation coefficient, within participants.

Pairwise correlations		Repeated measures correlation coefficient *r*_rm_	*p-*Value	Participants *n*			
Data category 1	Data category 2				Young only *r*_rm_ (*n*; *p*-value)	Male only *r*_rm_ (*n*; *p*-value)	BMI < 25 only *r*_rm_ (*n*; *p*-value)
Resting heart rate	Stand hours	−0.434	0.000	10	−0.442 (9; 0.001)	−0.438 (7; 0.002)	−0.028 (7; 0.878)
Resting heart rate	Heart rate variability	−0.439	0.000	14	−0.460 (11; 0.000)	−0.462 (10; 0.000)	−0.050 (10; 0.720)
Resting heart rate	VO_2_ max	−0.009	0.954	13			
Resting heart rate	Walking and running distance	−0.009	0.941	15			
Resting heart rate	Flights climbed	−0.05	0.646	17			
Resting heart rate	Step count	−0.003	0.979	17			
Stand hours	Heart rate variability	0.542	0.000	11	0.567 (10; 0.000)	0.591 (7; 0.000)	0.503 (7; 0.003)
VO_2_ max	Heart rate variability	0.215	0.103	14			
Walking and running distance	Heart rate variability	0.051	0.66	13			
Flights climbed	Heart rate variability	−0.083	0.444	15			
Step count	Heart rate variability	−0.032	0.764	15			

### Investigation of relationships between daily RHR, daily SH and daily HRV

**Figure 2.**
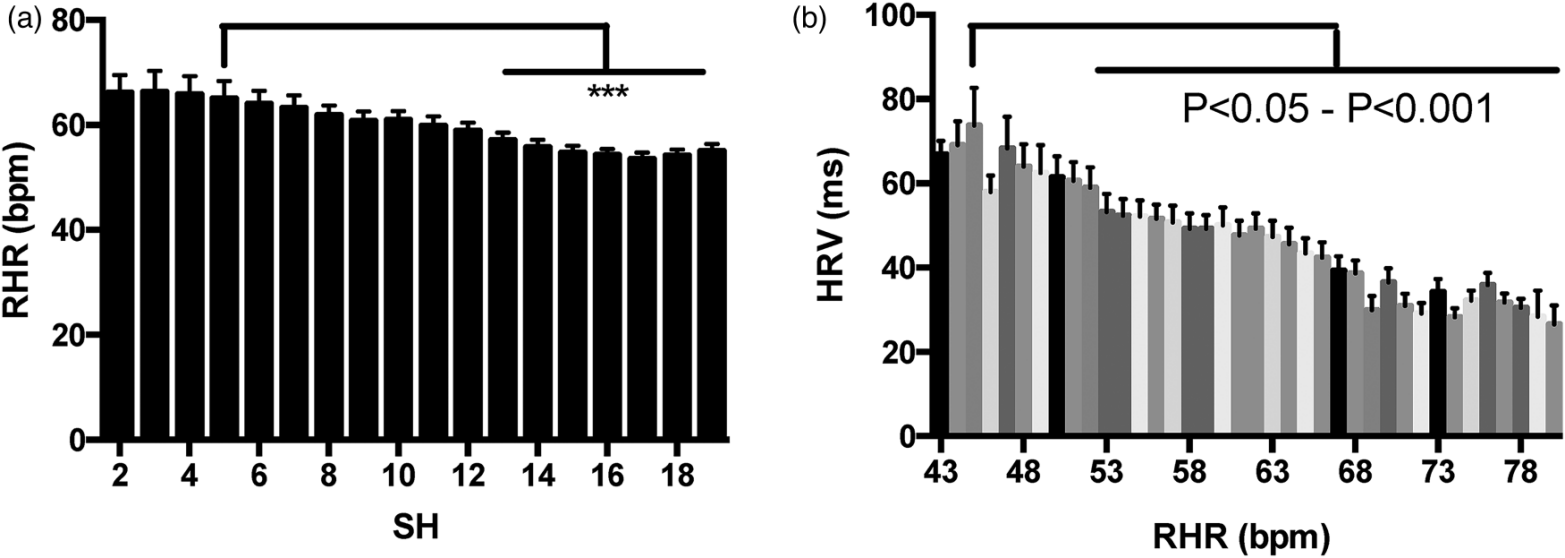
Histograms describing relationships between Standing Hours, Heart Rate Variability and Resting Heart Rate-daily values. Pairwise histograms are exhibited between (a) Resting Heart Rate and Stand Hours and (b) Heart Rate Variability and Resting Heart Rate. Pairwise statistical testing of selected higher and lower values in each histogram determined that the differences observed were significant (* is *p* < 0.05; ** is *p* < 0.01 and *** is *p* < 0.001).

We investigated whether differences in daily HRV and SH associated with differences in RHR in daily paired measurements. Plotting histograms, we discovered that between 2 and 17 SH, RHR was lower on days with higher SH, in a dose dependent manner (Figure 2(a)). A similar dose dependent effect was observed between HRV and RHR (Figure 2(b)). The relationship between SH and RHR is likely mediated by HRV, as HRV was higher when SH was higher (SH 5, HRV 40 ± 4 ms; SH 15, HRV 54 ± 4 ms, *p* = 0.03). These findings may be physiologically meaningful. On days with 15 SH, mean RHR was 10 beats per minute lower than on days with 5 SH (Figure 2(a)). Taken together with previous meta-analysis findings that an increase of 1 ms in lab-measured HRV (standard deviation of NN intervals, SDNN) reduces risk of CVD by ∼1%,^9^ the correlations we observe might suggest that promotion of desk breaks or other approaches such as standing desks, aimed at increasing frequency of standing throughout the day, merit prospective investigation for CVD prevention.

### Strengths and limitations of our study

The wearable devices that were used were participants’ own devices and were mostly Garmin and Apple watches, with one Polar watch. 15/20 of the wearables were Apple watches and two-thirds of the pair-wise analyses, including each one involving SH, HRV or VO_2_ max, do not contain any Garmin data. The data extraction strategy depended on the participant using an iPhone with iOS14 installed, so although the watch technology for data acquisition varied, the data storage platform was the same for each participant. Data recorded by the phone itself for categories including steps, WRD, FC and SH, will be present. Use of two devices to record activity will however have minimised periods of time when activity was not being monitored by any device, which is likely to have been of benefit, as we did not identify a satisfactory way to define and therefore adjust for non-wear time. We also collected demographic data as recommended.^15^

Regarding known limitations, our study could not be as well controlled as a lab-based one could have been; however, a key strength of our study utilising wearables is its ethnographic, in-community measurements outside an artificial lab environment that might itself act as a stressor affecting ANS poise (often referred to as the ‘white-coat’ effect^15^). In addition, the amounts of the daily data are far greater than generally can be achieved in a lab study, as also recognised previously.^15^ Our observational data does not demonstrate causality. As we studied only healthy, active individuals, based on convenience sampling, external validity, particularly for ‘at-risk’ patient groups has not been established and will need to be investigated in follow-up studies.

## Conclusion

By analysing high volume smartwatch data, our study finds that in the community, overall fitness and activity inversely correlates with RHR between participants. Frequency of standing (SH) and HRV both inversely correlate with RHR not only between participants but also *within participants*, suggesting they may particularly give individuals agency to modify their RHR. Our investigation provides community-based observational evidence supporting further prospective investigation of SH, in addition to more familiar interventions based on fitness and activity, as a potential CVD prevention strategy, through activities such as promotion of desk breaks. We recognise that observations made in existing watch owners will not necessarily translate to the patient groups who might benefit most from cardiac disease prevention interventions. The external validity for at-risk groups of these initial observations, should now therefore be tested through additional observational and interventional studies.
